# Multiple asymptomatic, hyperkeratotic minute filiform papules

**DOI:** 10.1016/j.jdcr.2024.01.008

**Published:** 2024-01-20

**Authors:** Sandra Jaroonwanichkul, Stephanie Matthews, Sarah Pourakbar, Hongyan Dai, Ting Wang-Weinman

**Affiliations:** a Department of Dermatology, University of Missouri – Kansas City School of Medicine, Kansas City, Missouri; b Division of Dermatology, University of Kansas Medical Center, Kansas City, Kansas; c Department of Pathology and Laboratory Medicine, University of Kansas Medical Center, Kansas City, Kansas

**Keywords:** breast cancer, chemotherapy, multiple minute digitate hyperkeratosis, papules, postirradiation digitate keratosis, phrynoderma, radiation, spiny keratoderma, trichodysplasia spinulosa

## Case

**Fig 1:**
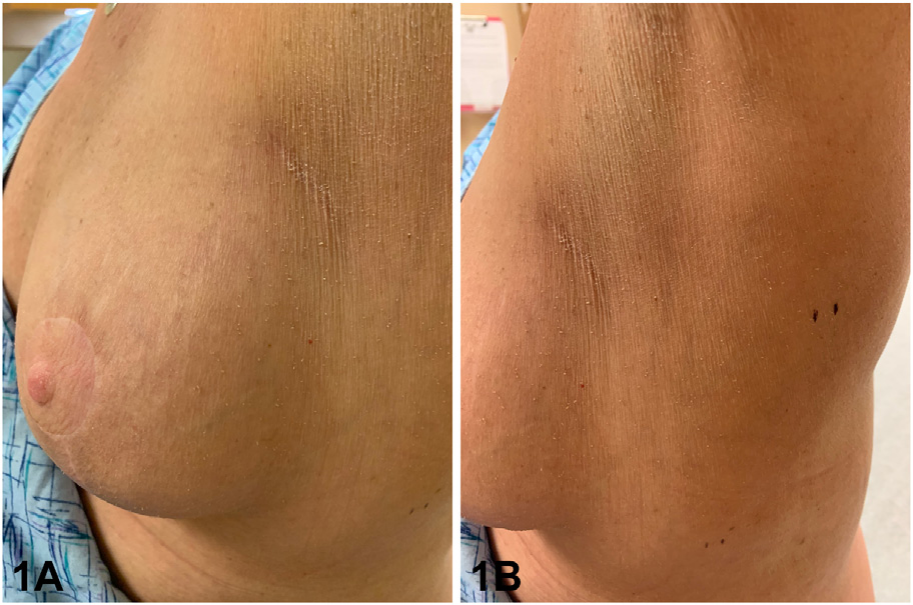

**Fig 2:**
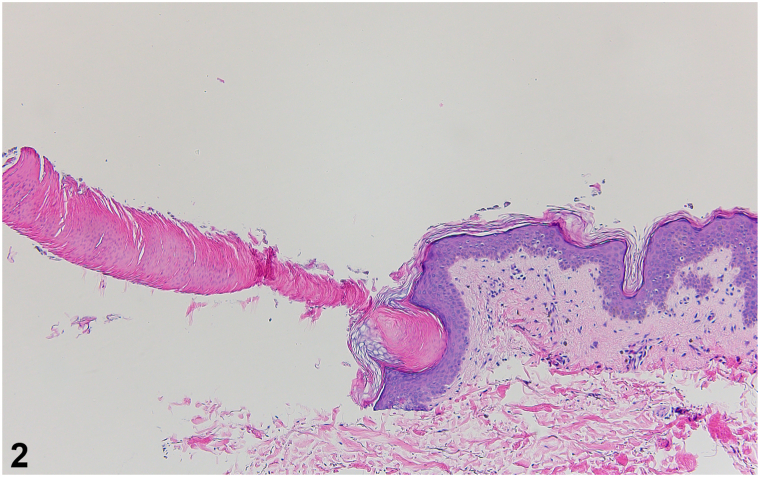

A 56-year-old woman with a history of left breast cancer treated with lumpectomy, radiation, and chemotherapy presented with multiple asymptomatic keratotic lesions around her left breast which had emerged 6 months prior to presentation. She completed radiotherapy to the left chest 1 year prior and noticed the lesions 3 to 4 months afterward. On examination, she had numerous thin 1 mm tall hyperkeratotic minute filiform papules scattered around the left breast, extending to the left lateral breast and inferior axilla, comprising an area limited to the field of radiation (Fig 1, *A* and *B*). A punch biopsy was obtained for further evaluation (Fig 2).

**Question 1: What is the most likely diagnosis?**

A. Postirradiation digitate keratosis (PIDK)
B. Multiple minute digitate hyperkeratosis (MMDH)
C. Trichodysplasia spinulosa
D. Spiny keratoderma
E. Phrynoderma

**Answers:**

A. Postirradiation digitate keratosis (PIDK) – Correct. PIDK is a rare porokeratotic skin condition clinically characterized by multiple asymptomatic, hyperkeratotic minute filiform papules developing months to years following radiotherapy, with findings mostly confined to the field of radiation. It is histologically characterized by cornoid lamellae, nonfollicular focal columns of parakeratotic hyperkeratosis, over an atrophic, invaginated epidermis devoid of a granular layer.
B. Multiple minute digitate hyperkeratosis (MMDH) – Incorrect. MMDH clinically presents similar to PIDK, with hyperkeratotic minute filiform projections and flat-topped to dome-shaped papules. However, the distribution of the lesions is generalized rather than limited to the field of radiation, with predilection for the trunk and proximal extremities. Histopathologic findings include nonfollicular focal columns of orthokeratotic hyperkeratosis with a tented epidermis and slight acanthosis. In rare cases, MMDH presents similarly to PIDK on histology, demonstrating nonfollicular focal columns of parakeratotic hyperkeratosis and underlying epidermal invagination.^1^ Patient history and distribution of the lesions are essential distinguishing features in these circumstances.
C. Trichodysplasia spinulosa – Incorrect. Trichodysplasia spinulosa occurs secondary to polyomavirus infection and is predominantly seen in immunosuppressed individuals. It clinically presents with follicular keratotic spiny papules on the face. Histopathology reveals follicular association of the spines, and follicles contain enlarged trichohyalin granules. PIDK is a nonfollicular associated condition.
D. Spiny keratoderma – Incorrect. In spiny keratoderma, filiform papules appear on palmoplantar surfaces. Histopathology is variable, demonstrating orthokeratotic or parakeratotic hyperkeratosis with a tented or invaginated epidermis.
E. Phrynoderma – Incorrect. Phrynoderma is associated with nutritional deficiencies and exhibits follicular spines in a generalized distribution. Histopathologic findings include follicular hyperkeratosis.

**Question 2: What is the most commonly associated predisposing malignancy for this condition?**

A. Angiosarcoma
B. Breast cancer
C. Multiple myeloma
D. Squamous cell carcinoma
E. Bronchial carcinoma

**Answers:**

A. Angiosarcoma – Incorrect. Angiosarcoma is not a commonly associated predisposing malignancy for PIDK. However, there have been several cases of radiation-induced angiosarcoma arising following treatment with radiotherapy for breast cancer.^2^
B. Breast cancer – Correct. All previously reported cases of PIDK have occurred in women with a history of breast cancer treated with mastectomy and radiotherapy. The time frame from radiation to the appearance of minute filiform projections ranges from 9 months to 5 years. In the current case, rapid clinical appearance of papules emerged in as little as 3 to 4 months. PIDK is a diagnosis to strongly consider in patients who present with multiple asymptomatic minute filiform papules in sites of previous radiotherapy for breast cancer.
C. Multiple myeloma – Incorrect. Both MMDH and spiny keratoderma, not PIDK, have been observed to be associated with multiple myeloma.^3^^,^^4^
D. Squamous cell carcinoma – Incorrect. Squamous cell carcinoma is not a commonly associated predisposing malignancy for PIDK. Since PIDK is a form of porokeratosis, there is a risk of malignant transformation to squamous cell carcinoma or basal cell carcinoma. Thus, patients should be instructed to follow up for malignancy surveillance.
E. Bronchial carcinoma – Incorrect. Bronchial carcinoma has been described to be associated with MMDH and spiny keratoderma, not PIDK.^3^^,^^4^

**Question 3: Which of the following is the most appropriate treatment option for this condition?**

A. Topical cidofovir
B. Nutritional supplementation
C. Topical alpha-hydroxy or beta-hydroxy moisturizer
D. Oral valganciclovir
E. Oral doxycycline

**Answers:**

A. Topical cidofovir – Incorrect. One percent or 3% topical cidofovir is an effective treatment option for trichodysplasia spinulosa, not PIDK.
B. Nutritional supplementation – Incorrect. Nutritional supplementation would be warranted in cases of phrynoderma.
C. Topical alpha-hydroxy or beta-hydroxy moisturizer – Correct. Due to the asymptomatic nature of PIDK lesions, treatment is optional per patient preference. Treatment includes a topical alpha-hydroxy or beta-hydroxy moisturizer, a keratolytic that alters corneocyte cohesion, leading to exfoliation of the hyperkeratosis. It has been postulated that treatments used for porokeratosis including topical vitamin D analogs, retinoids, and topical lovastatin/cholesterol could be trialed in refractory cases; 2% salicylic acid ointment was prescribed in 1 case which led to the resolution of the condition.^5^ Our patient failed to respond to topical tretinoin, 6% salicylic acid cream, and topical alpha-hydroxy moisturizer. Subsequently, she elected to monitor.
D. Oral valganciclovir – Incorrect. Oral valganciclovir would be an effective treatment option for trichodysplasia spinulosa, not PIDK.
E. Oral doxycycline – Incorrect. PIDK is not an infectious process and, thus, would not necessitate antibiotic therapy.

## Conflicts of interest

None disclosed.
